# TLR3 signaling-induced interferon-stimulated gene 56 plays a role in the pathogenesis of rheumatoid arthritis

**DOI:** 10.3389/ebm.2024.10122

**Published:** 2024-05-31

**Authors:** Hikaru Kristi Ishibashi, Yuzuru Nakamura, Tatsuro Saruga, Tadaatsu Imaizumi, Akira Kurose, Shogo Kawaguchi, Kazuhiko Seya, Eiji Sasaki, Yasuyuki Ishibashi

**Affiliations:** ^1^ Department of Orthopaedic Surgery, Hirosaki University Graduate School of Medicine, Hirosaki, Aomori, Japan; ^2^ Department of Vascular Biology, Hirosaki University Graduate School of Medicine, Hirosaki, Aomori, Japan; ^3^ Department of Anatomic Pathology, Hirosaki University Graduate School of Medicine, Hirosaki, Aomori, Japan

**Keywords:** rheumatoid arthritis, ISG56, CXCL10, TLR3, synovitis

## Abstract

Rheumatoid fibroblast-like synoviocytes (RFLS) have an important role in the inflammatory pathogenesis of rheumatoid arthritis (RA). Toll-like receptor 3 (TLR3) is upregulated in RFLS; its activation leads to the production of interferon-β (IFN-β), a type I IFN. IFN-stimulated gene 56 (ISG56) is induced by IFN and is involved in innate immune responses; however, its role in RA remains unknown. Therefore, the purpose of this study was to investigate the role of TLR3-induced ISG56 in human RFLS. RFLS were treated with polyinosinic-polycytidylic acid (poly I:C), which served as a TLR3 ligand. ISG56, melanoma differentiation-associated gene 5 (MDA5), and C-X-C motif chemokine ligand 10 (CXCL10) expression were measured using quantitative reverse transcription-polymerase chain reaction, western blotting, and enzyme-linked immunosorbent assay. Using immunohistochemistry, we found that ISG56 was expressed in synovial tissues of patients with RA and osteoarthritis. Under poly I:C treatment, ISG56 was upregulated in RFLS. In addition, we found that the type I IFN-neutralizing antibody mixture suppressed ISG56 expression. ISG56 knockdown decreased CXCL10 expression and MDA5 knockdown decreased ISG56 expression. In addition, we found that ISG56 was strongly expressed in the synovial cells of patients with RA. TLR3 signaling induced ISG56 expression in RFLS and type I IFN was involved in ISG56 expression. ISG56 was also found to be associated with CXCL10 expression, suggesting that ISG56 may be involved in TLR3/type I IFN/CXCL10 axis, and play a role in RA synovial inflammation.

## Impact statement

In human mesangial cells, the interferon (IFN)-stimulated gene 56 (ISG56) is induced by Toll-like receptor 3 (TLR3) signaling and is involved in innate inflammatory responses. The role of ISG56 in rheumatoid fibroblast-like synoviocytes (RFLS) remains unclear. Therefore, we evaluated ISG56 expression in cultured human RFLS using the TLR3 agonist. Under poly I:C treatment, ISG56 expression was upregulated in RFLS, and a type I IFN-neutralizing antibody mixture suppressed ISG56 expression. ISG56 knockdown decreased CXCL10 expression. ISG56 was strongly expressed in the synovial cells of patients with RA. TLR3 signaling induced ISG56 expression in RFLS and type I IFN was involved in ISG56 expression. These findings suggest that ISG56 is involved in the TLR3/type I IFN/CXCL10 axis, which plays an important role in the inflammatory responses in RFLS. Thus, ISG56 may be a potential target for the development of novel RA treatments.

## Introduction

Rheumatoid arthritis (RA) is a systemic autoimmune disease that causes chronic inflammation of synovial joints as well as the cartilage and bones within the joint primarily. Fibroblast-like synoviocytes (FLS) are important in the pathogenesis of RA because FLS produce inflammatory cytokines and proteases that contribute to cartilage destruction [1]. Therefore, in recent years, RA therapies have focused on FLS targeting [2, 3]. Toll-like receptors (TLRs) are pattern recognition receptors (PRRs), present in various cells and tissues [4], capable of recognizing pathogen-associated molecular patterns (PAMPs; expressed by microbes) and danger-associated molecular patterns (DAMPs; expressed by dead or injured cells). TLR3 detects double-stranded RNA (dsRNA) to activate innate immune responses. In the joint fluid of RA patients, dsRNA levels are elevated [5], suggesting that dsRNA functions as DAMPs in RFLS. Activation of TLRs, including TLR3, in synoviocytes aggravates arthritis [6]. Therefore, understanding the contribution of TLR3 signaling in RFLS could provide a basis for the development of new therapeutic interventions. However, the molecular mechanisms underlying TLR3-mediated inflammation in RFLS have not been fully elucidated. The binding of dsRNA to TLR3 leads to the production of a type I interferon (IFN), IFN-β. IFN-β is a key cytokine in antiviral immunity. IFNs exert their biological effects by inducing the expression of hundreds of IFN-stimulated genes (ISGs). ISGs encode several chemokines which are small molecular proteins that induce the chemotactic activity of leukocytes, leading to pro-inflammatory reactions. An ISG example is the C-X-C motif chemokine ligand 10 (CXCL10). CXCL10 expression is upregulated in serum and tissue of patients with RA [7]. Owing to its role in leukocyte homing to inflamed tissues and the persistence of inflammation, CXCL10 upregulation may contribute significantly to tissue damage. In addition to CXCL10, other ISGs may contribute to rheumatoid synovial inflammation. Melanoma differentiation-associated gene 5 (MDA5) is a type Ⅰ IFN-induced ISG in melanoma cells [8]. MDA5 is a DExH group RNA helicase; it functions as an ATPase and a signaling molecule that regulates inflammatory responses [9]. Saruga et al. [10] showed that the TLR3/type Ⅰ IFN/CXCL10 axis plays an important role in the RFLS inflammatory response. In addition, MDA5 may be associated with this axis. Another ISG, ISG56 (official gene symbol: interferon-induced protein with tetratricopeptide repeat (IFIT) 1) is induced by dsRNA, type Ⅰ IFN, and viruses [11]. It is associated with the innate immune response [12]. In addition, ISG56 is involved in immune and inflammatory responses induced by TLR3 signaling in human mesangial [9] and astrocytoma cells [13]. However, the expression and role of ISG56 in RFLS remains unknown. We hypothesized that ISG56 plays a role in rheumatoid synovitis downstream of TLR3. Therefore, in the current study, we aimed to investigate the expression and role of ISG56 in RFLS, using the TLR3 agonist polyinosinic-polycytidylic acid (poly I:C). In addition, we investigated the relationship between ISG56 and the TLR3/type Ⅰ IFN/CXCL10 axis. In addition, we evaluated the expression of the ISG56 protein in the tissues of patients with RA.

## Materials and methods

### Cell culture

Patient-derived human RFLS were purchased from the Health Science Research Resource Bank (Sennan, Japan). The cells were cultured in Dulbecco’s modified Eagle’s medium supplemented with 10% fetal bovine serum and treated with 0.4–50 μg/mL poly I:C (Sigma-Aldrich, St. Louis, MO, United States). The cells were incubated for up to 24 h at 37°C. For experiments performed with the anti-human type I IFN-neutralizing antibody mixture (PBL Assay Science, Piscataway, NJ, United States), the cells were preincubated with the antibody mixture (1:25 dilution) for 1 h and stimulated with 10 μg/mL poly I:C for 16 h. For RNA interference, RFLS were transfected with non-silencing control small interfering RNA (siRNA) and siRNA against ISG56 or MDA5 (Qiagen, Hilden, Germany) using the Lipofectamine RNAiMAX reagent (ThermoFisher, Waltham, MA, United States), according to the manufacturer’s instructions. The transfected cells were incubated for 48 h at 37°C. Subsequently, the cells were treated with poly I:C. This study was approved by the Hirosaki University Graduate School of Medicine Ethics Committee (2018-1117) [10]. The sequences of siRNAs are as follows.

ISG56 sense strand; 5′-GGC​UGU​CCG​CUU​AAA​UCC​ATT-3′,

ISG56 antisense strand; 5′-UGG​AUU​UAA​GCG​GAC​AGC​CTG-3′,

MDA5 sense strand; 5′-GGU​GUA​AGA​GAG​CUA​CUA​ATT-3′, and MDA5 antisense strand; 5′-UUA​GUA​GCU​CUC​UUA​CAC​CTG-3′.

### Quantitative reverse transcription-polymerase chain reaction (qRT-PCR)

Total RNA was extracted from incubated cells and purified using the illustraRNA spin kit (GE healthcare, Buckinghamshire, England). Using the purified RNA product as the template and the oligo(dT)_18_ primer and Moloney Murine Leukemia Virus reverse transcriptase (ThermoFisher), single-stranded cDNA was synthesized. The following primers were used for cDNA amplification:

TLR3-F: 5′- CTC​AGA​AGA​TTA​CCA​GCC​GCC -3′,

TLR3-R: 5′- CCA​TTA​TGA​GAC​AGA​TCT​AAT​G -3′,

ISG56-F: 5′-TAG​CCA​ACA​TGT​CCT​CAC​AGA​C-3′,

ISG56-R: 5′-TCT​TCT​ACC​ACT​GGT​TTC-A-T-GC-3′,

CXCL10-F: 5′-TTC​AAG​GAG​TAC​CTC​TCT​CTA​G-3′,

CXCL10-R: 5′-CTG​GAT​TCA​GAC-A-T-CTC​TTC​TC-3′,

MDA5-F: 5′-GTT​GAA​AAG​GCT​GGC​TGA​AAA​C-3′

MDA5-R: 5′-TCG​ATA​ACT​CCT​GAA​CCA​CTG-3′

GAPDH-F: 5′-GCA​CCG​TCA​AGG​CTG​AGA​AC-3′,

GAPDH-R: 5′-ATG​GTG​GTG​AAG​ACG​CCA​GT-3′.

To quantify ISG56, CXCL10, and MDA5 mRNA expression, a real-time PCR system was utilized with the SsoAdvanced Universal SYBR Green Supermix (Bio-Rad, Hercules, CA, United States). GAPDH was used as an internal control. ISG56 and MDA5 expression were each represented as a fold change relative to that in untreated cells. CXCL10 expression was below the CXCL10 expression detection limit in untreated cells; therefore, the data was expressed in arbitrary units.

### Western blotting

Cultured cells were washed and lysed in Laemmli sample buffer. Cell lysates were subjected to electrophoretic separation using polyacrylamide gel electrophoresis. Then, the proteins were transferred to polyvinylidene fluoride membranes. The membranes were blocked and incubated, with rabbit antibodies against TLR3 (Cell Signaling Technology, Danvers, MA, United States; 1:1000 dilution), IFIT1/ISG56 (Genetex, Irvine, CA, United States; 1:2500 dilution), MDA5 (Immuno-Biological Laboratories, Gunma, Japan; 1:2000 dilution), or actin (Sigma-Aldrich; 1:5,000 dilution), overnight at 4°C. ISG56 and actin protein signals were obtained using a horseradish peroxidase (HRP)-conjugated anti-rabbit IgG gout antibody (Medical and Biological Laboratories, Tokyo, Japan; 1:10,000 dilution) and an HRP chemiluminescent substrate. The bands of western blotting were quantified with ImageJ (National Institute of Health, United States).

### Enzyme-linked immunosorbent assay (ELISA)

The cell culture medium was collected and centrifuged. The CXCL10 protein concentration, in the supernatant, was determined using a sandwich ELISA kit (R&D systems, Minneapolis, MN, United States).

### Statistics

Differences between groups were analyzed using the Student’s t-tests. Data inputs and analyses were performed using SPSS version 29.0 (SPSS Inc., Chicago, IL, United States). Statistical significance was set at *p*-value < 0.05.

### Immunohistochemical analysis

To determine ISG56 expression in human rheumatoid synovial tissue, we obtained knee synovial tissues from four RA and four osteoarthritis (OA) patients who had undergone total knee arthroplasty. Synovial tissues from patients with knee OA were used as controls. The obtained tissue samples were fixed in formalin, paraffin-embedded, and sliced into 3 μm thick sections. Sections were probed with rabbit anti-ISG56/IFIT1 polyclonal antibody (1:50, GTX31570, GeneTex) and incubated with biotinylated anti-rabbit IgG antibody and HRP-conjugated streptavidin. The ISG56 protein was detected using 3,3′-Diaminobenzidine, and the protein intensity was measured by a pathologist with more than 20 years of experience.

The study was performed in accordance with the 1964 Declaration of Helsinki and its later amendments or comparable ethical standards. Approval was obtained from the ethics committee of Hirosaki University Graduate School of Medicine (2018-1117). Written informed consent was obtained from all the participants.

## Results

### TLR3 was expressed in RFLS

**FIGURE 1:**
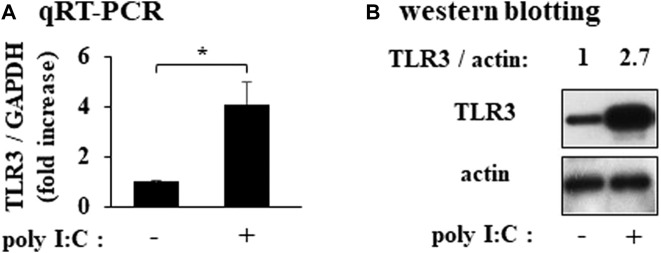
Expression of TLR3 in cultured human rheumatoid fibroblast-like synoviocytes (RFLS) incubated with polyinosinic-polycytidylic acid (poly I:C) **(A)** Cultured RFLS were treated with 10 μg/mL poly I:C for 8 h. After RNA extraction, cDNA was synthesized, and quantitative real-time PCR was performed to amplify TLR3 and GAPDH cDNA.TLR3 mRNA expression was normalized to that of GAPDH. Data for TLR3 are shown as fold increase relative to that of unstimulated cells. **(B)** Cells were treated with poly I:C and lysed after 16 h incubation. The lysates were subjected to western blotting to determine TLR3 and actin protein expression. *p < 0.01, by Student’s t-test.

In cultured RFLS, TLR3 mRNA and protein were expressed and the expression were increased by treatment with poly I:C (Figures 1A, B).

### Poly I:C induces the expression of ISG56 in cultured RFLS

**FIGURE 2:**
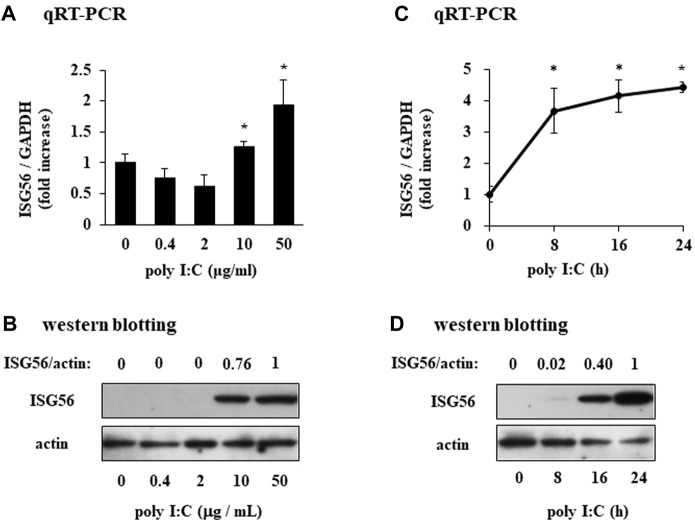
Expression of ISG56 in cultured human RFLS incubated with poly I:C **(A)** RFLS were cultured and treated with 0.4–50 μg/mL poly I:C. After 8 h of incubation, RNA was extracted, cDNA was synthesized, and quantitative real-time PCR was performed to amplify ISG56 and GAPDH cDNA. ISG56 mRNA expression was normalized to that of GAPDH. Data for ISG56 are shown as fold increase relative to that of unstimulated cells. **(B)** Cells were treated with poly I:C and lysed 16 h post-incubation. The lysates were subjected to western blotting to determine ISG56 and actin protein expression. **(C,D)** Time course of ISG56 mRNA and protein expression in RFLS incubated with poly I:C. The cells were treated with 30 μg/mL poly I:C for up to 24 h, and RNA and protein were obtained. ISG56 mRNA and protein expression was measured using qRT-PCR **(C)** and western blotting **(D)**, respectively. Data in **(A,C)** represent the mean ± standard deviation (*n* = 3). *p < 0.01, by Student’s t-test.

In unstimulated RFLS, ISG56 expression was weak and the protein was undetectable (Figures 2A, B). In RFLS stimulated with low poly I:C concentrations (0.4 and 2 μg/mL), ISG56 mRNA was not upregulated. However, higher poly I:C concentrations (10 and 50 μg/mL) induced ISG56 mRNA (Figure 2A) and protein (Figure 2B) expression in RFLS. ISG56 mRNA and protein expression increased–8–24 h and, 16–24 h after poly IC treatment, respectively (Figures 2C, D).

### Type I IFN is implicated in the expression of ISG56 under poly I:C treatment

**FIGURE 3:**
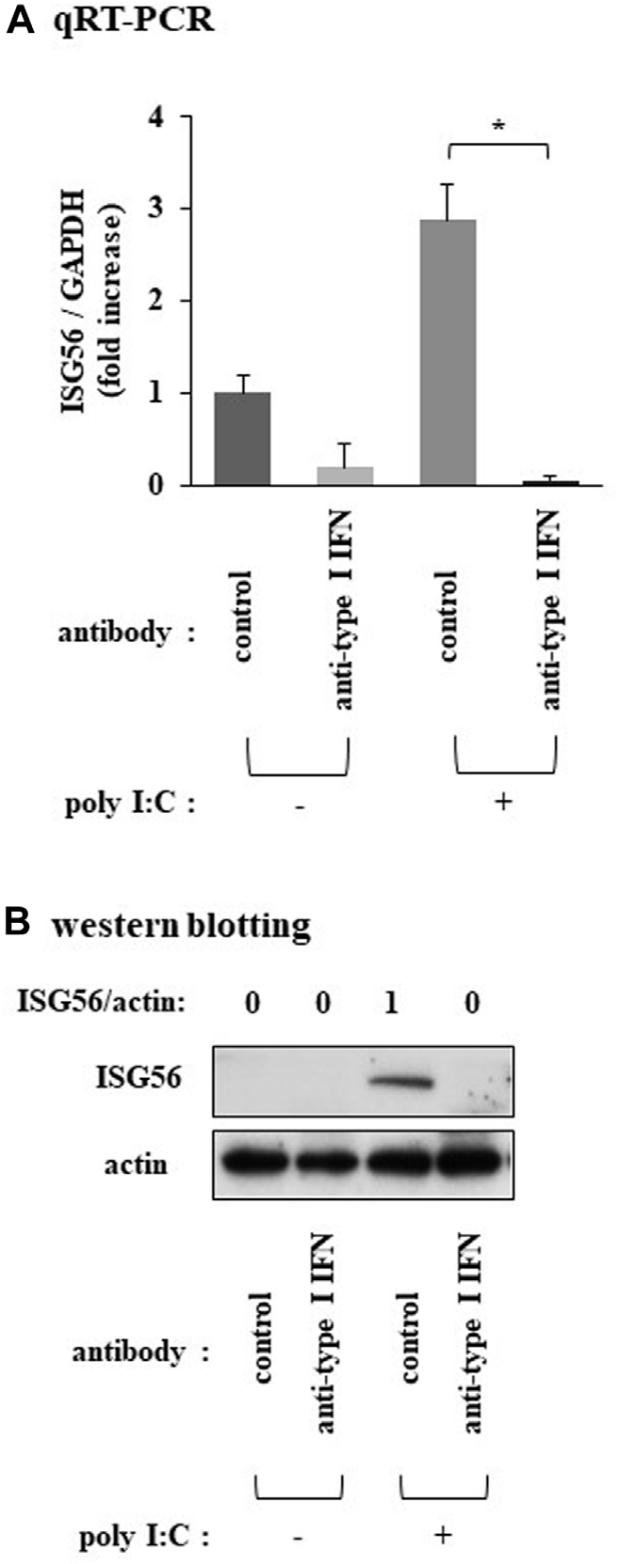
Expression of ISG56 under treatment with a neutralizing type I IFN antibody mixture in RFLS incubated with poly I:C Cells were preincubated with human type I IFN-neutralizing antibody mixture (1:25 dilution) for 1 h and further treated with 30 μg/mL poly I:C for 16 h. **(A)** RNA was extracted and qRT-PCR was performed. **(B)** Cells were lysed and ISG56 and actin protein expression were determined using western blotting. Data in **(A)** represent the mean ± standard deviation (*n* = 3). *p < 0.01, by Student’s t-test.

Pretreatment of cells with a neutralizing anti-type I IFN antibody mixture almost completely reduced the poly I:C-mediated induction of ISG56 mRNA (Figure 3A) and protein (Figure 3B).

### ISG56 positively regulates the expression of CXCL10 under poly I:C treatment

**FIGURE 4:**
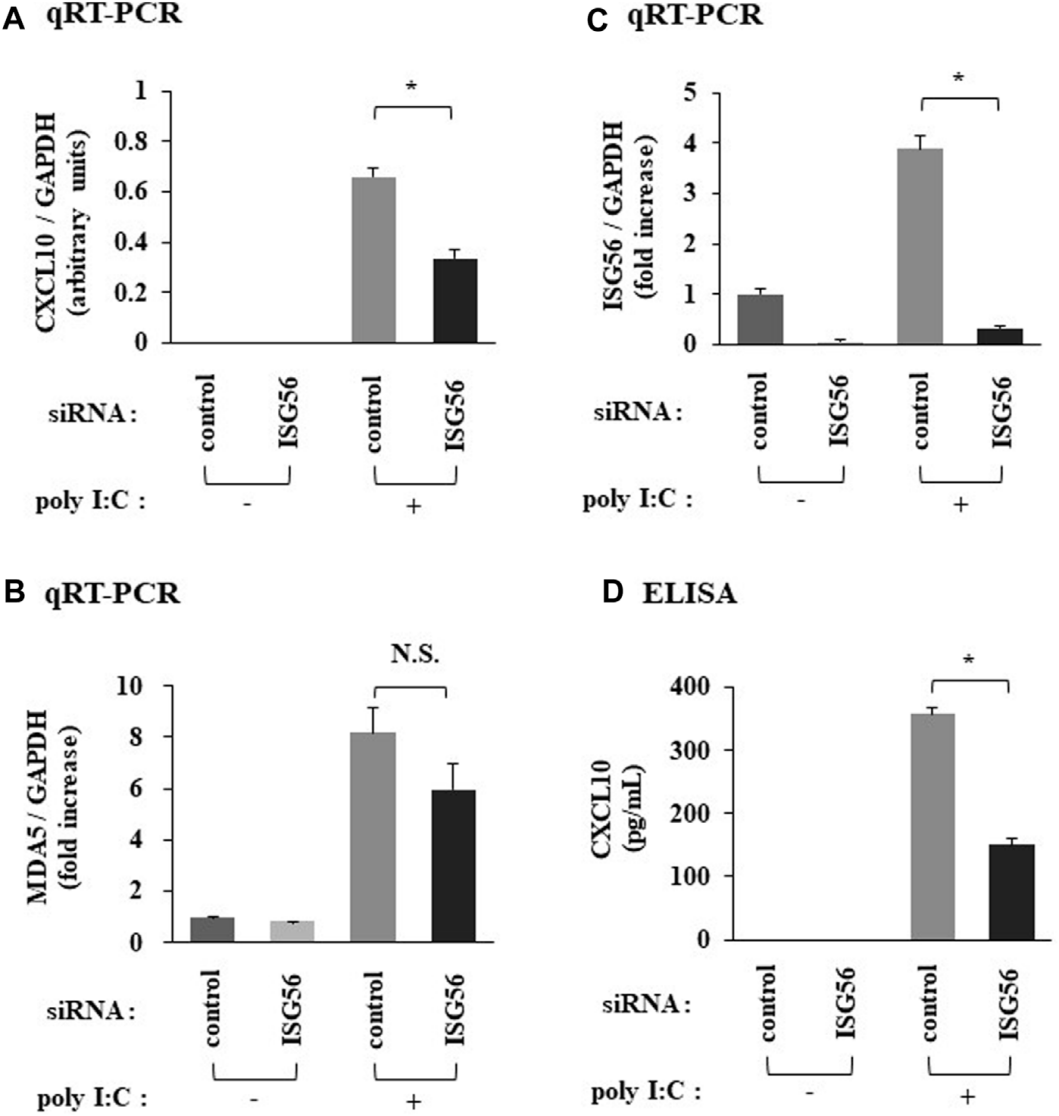
Expression of CXCL10 and MDA5 in poly IC-treated RFLS under the introduction of small interfering RNA (siRNA) against ISG56 Cells were transfected with siRNA against ISG56 and incubated for 48 h. Next, cells were treated with 30 μg/mL poly I:C for another 16 h. RNA was extracted and qRT-PCR was performed to determine CXCL10 **(A)**, MDA5 **(B)**, and ISG56 **(C)** mRNA expression. **(D)** The medium was collected and subjected to CXCL10 ELISA. Data from **(A–D)** represent the mean ± standard deviation (*n* = 3). *p < 0.01 by Student’s t-test. NS, not significant by Student’s t-test.

Transfection of cells, under poly I:C treatment, with siRNA against ISG56 significantly reduced CXCL10 mRNA (Figure 4A) and protein (Figure 4D) expression. In ISG56 knockdown cells poly I:C did not significantly induce MDA5 mRNA expression (Figure 4B). ISG56 knockdown was confirmed using qRT-PCR (Figure 4C).

### MDA5 positively mediates the expression of ISG56 under poly I:C treatment

**FIGURE 5:**
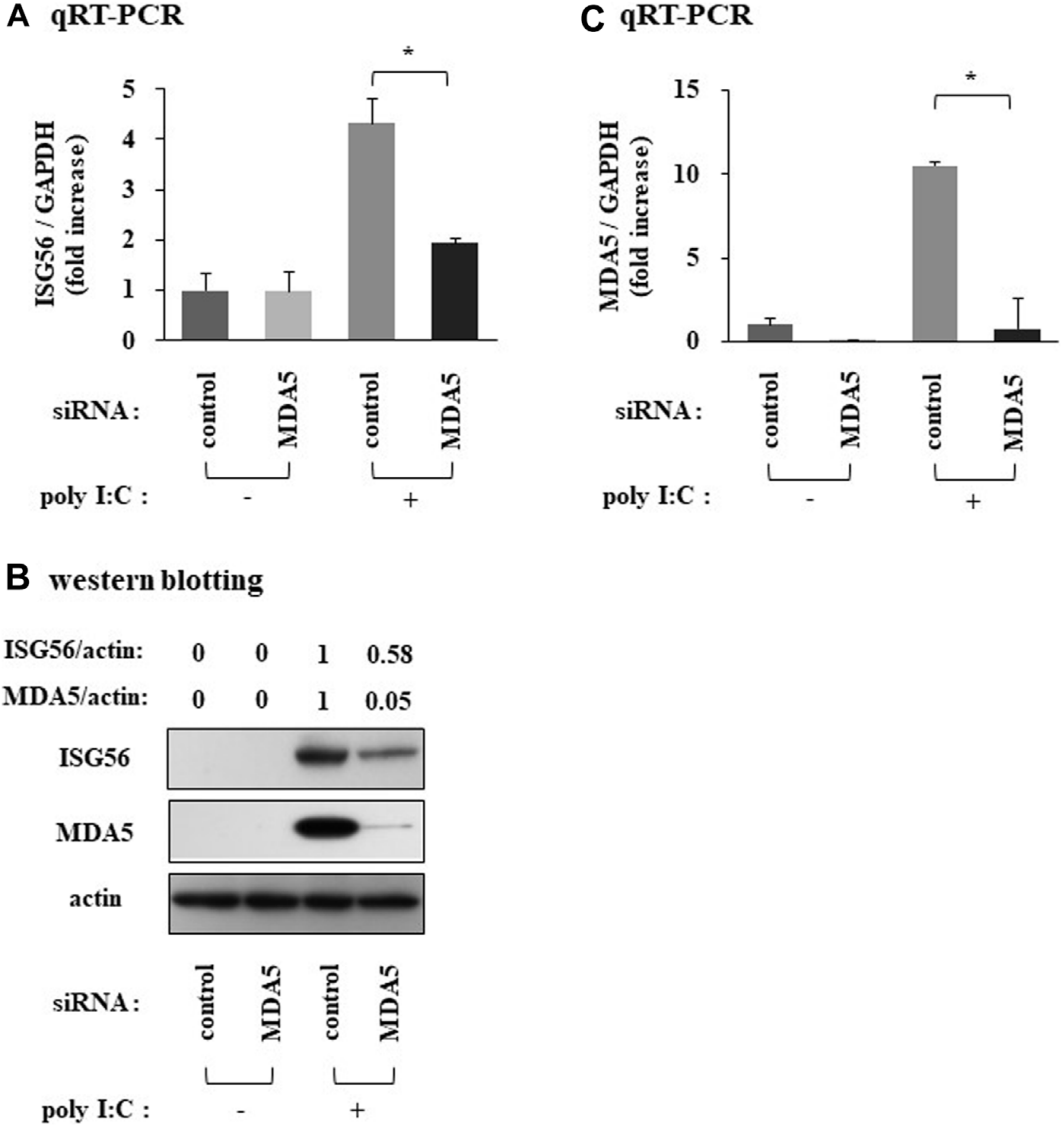
Expression of ISG56 under treatment with siRNA against MDA5 in RFLS Cells were transfected with siRNA against MDA5 and incubated for 48 h. Next, cells were treated with 30 μg/mL poly **I**:C for 16 h. RNA was extracted and qRT-PCR was performed to determine ISG56 **(A)** and MDA5 **(C)** mRNA expression. **(B)** Cells were lysed; ISG56, MDA5 and actin protein expression were measured using western blotting. Data from **(A**,**C)** represent the mean ± standard deviation (*n* = 3). *p < 0.01, by Student’s t-test.

SiRNA against MDA5 reduced the poly I:C-induced ISG56 mRNA (Figure 5A) and protein (Figure 5B) expression. MDA5 knockdown was confirmed using qRT-PCR and western blotting (Figures 5B, C).

### ISG56 expression is upregulated in RA synovial cells

**FIGURE 6:**
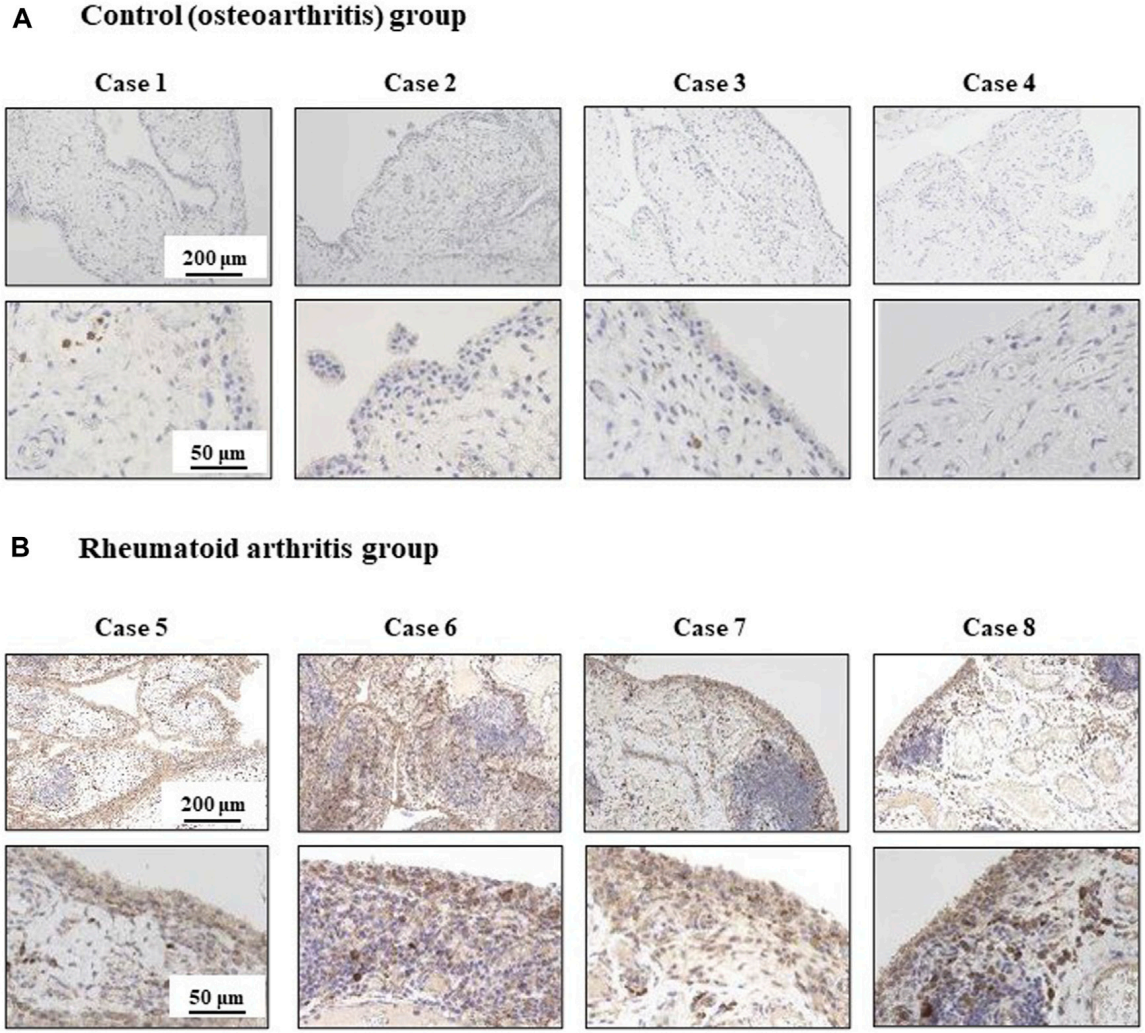
Immunohistochemical analysis for ISG56 in knee synovial tissue from osteoarthritis and rheumatoid arthritis patients Synovial tissue was collected from patients that underwent total knee arthroplasty. This included patients with osteoarthritis (OA) [cases 1-4, *n* = 4, **(A)**] and rheumatoid arthritis (RA) [cases 5-8, *n* = 4, **(B)**]. ISG56 expression was examined by immunohistochemistry. The immunoreactivity of ISG56 was stronger in RA synovial cells than in OA synovial cells.

The immunohistochemistry results are shown in Figure 6. In the tissues of patients with OA, ISG56 immunoreactivity was positive in plasma cells, but negligible in synovial cells (case 1-4). In contrast, intense ISG56 immunoreactivity was observed in both the plasma and synovial cells of patients with RA (case 5–8).

## Discussion

Multiple genetic and environmental factors are associated with the development of RA. Upon RA onset, innate and adaptive immunity are activated; as a result, a variety of immunoregulatory and proinflammatory molecules are expressed [14]. FLS are an important cell type in RA pathogenesis [15]; therefore, elucidating the role of FLS in the pathogenesis of RA may provide important insights for developing new RA therapies. In this study, we evaluated the expression of ISG56, an IFN-inducible molecule involved in innate immunity, in cultured human RFLS. Here, we report that the treatment of RFLS with poly I:C, a TLR3 agonist, induced ISG56 expression. In addition, we showed that the neutralizing type I IFN antibody mixture inhibited poly IC-induced ISG56 expression, indicating that ISG56 may be induced in type I IFN-dependent manner. The involvement of TLR3 signaling in the pathogenesis of RA has been previously reported [16]; however, this is the first study that highlights the role of ISG56 in RFLS.

ISG56 is a member of the IFIT RNA-binding protein family. IFIT proteins function as anti-viral molecules via multiple mechanisms, such as inhibiting the translation of non-self RNA, during viral infection [17]. In addition to their antiviral functions, IFIT proteins are also associated with inflammation. ISG56 is associated with autoimmune diseases, such as systemic lupus erythematosus [18] and lupus nephritis [19, 20]. Therefore, the results of the present study strongly suggests that ISG56 is associated with RA pathogenesis.

Cytokines produced by FLS bind to their receptors, activate signaling cascades, and mediate inflammatory responses, leading to synovitis, cartilage, and bone destruction [1, 21]. Chemokines induce leukocyte chemotaxis; and chemokines as well as chemokine receptors play a pathogenic role in RA [22]. CXCL10, a chemokine, contributes to the pathogenesis of autoimmune diseases [23]. CXCL10 expression is higher in the synovial fluid and serum of patients with RA [7, 24]. In patients with RA, CXCL10 upregulates the receptor activator of nuclear factor-B ligand (RANKL), which is involved in bone destruction [25]. In addition, a human phase II clinical trial showed that an anti-CXCL10 monoclonal antibody significantly improved the American College of Rheumatology 20% improvement criteria [26]. These findings suggest that CXCL10 plays an important role in the pathogenesis of RA and that it is important to clarify the mechanisms underlying the role of CXCL10 in RFLS. In RFLS treated with poly I:C, CXCL10 is produced via the TLR3/type I IFN/CXCL10 axis; a reaction that also involves MDA5 (another ISG) [10]. In this study, we found that ISG56 knockdown decreases the expression of CXCL10, but not MDA5. In addition, we found that MDA5 knockdown decreases ISG56 expression. These results suggest that ISG56 may be involved in MDA5-mediated CXCL10 expression in RFLS and CXCL10-mediated synovial inflammation in rheumatic joints. Although the precise molecular mechanisms are not yet clear, the study results indicate that a complex network of ISGs may be involved in the RFLS signaling pathways. Further investigations are required to elucidate the molecular mechanisms.

We also found that ISG56 was upregulated in synoviocytes of patients with RA using immunohistochemical analysis. In contrast, ISG56 expression in synoviocytes of patients with OA was negligible. This suggests that ISG56 plays a role in synovial inflammation in the joints of patients with RA.

The current study has several limitations. In the present study, we did not elucidate the precise molecular mechanism underlying ISG56-mediated CXCL10 expression in poly I:C-treated RFLS. However, the immunohistochemistry results demonstrated a clear difference in ISG56 expression between patients with RA and OA. A small sample size was used. In addition, experiments using animal models have not yet been conducted. Therefore, further research is necessary to verify the results of the study.

## Conclusion

**FIGURE 7:**
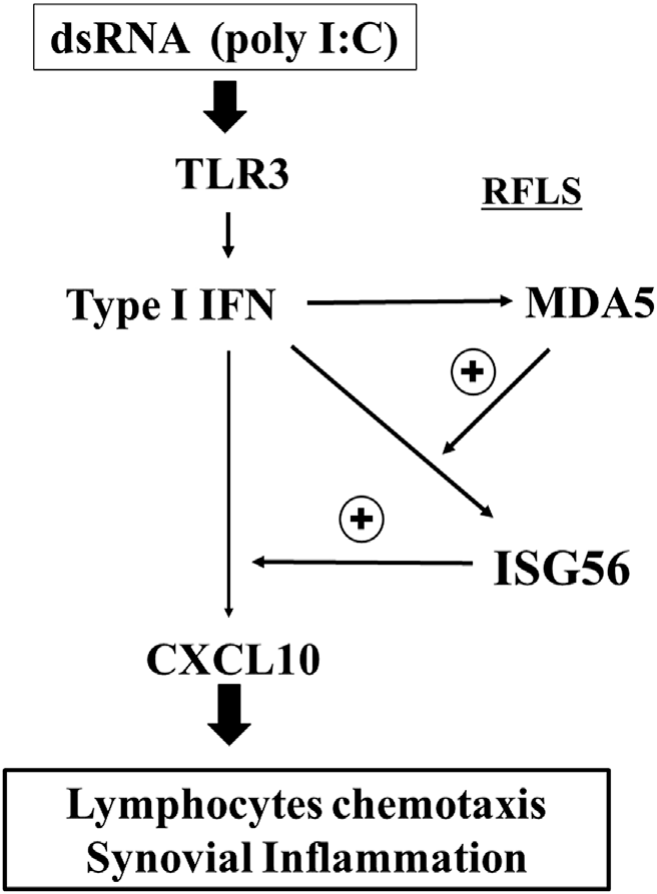
Predicted role of ISG56 expression via TLR3 in RFLS TLR3 activation by poly I:C induces the expression of CXCL10 in RFLS. CXCL10 induction is positively regulated by ISG56, and ISG56 expression is upregulated by type I IFN and positively regulated by MDA5. CXCL10 induces lymphocyte chemotaxis followed by synovial inflammation. Thus, ISG56 may be involved in rheumatoid synovial inflammation, at least in part, by enhancing TLR3-mediated CXCL10 expression.

This study showed that ISG56 expression is induced in cultured RFLS via the TLR3/type I IFN axis. In addition, it was shown that ISG56 may positively regulate CXCL10 expression, induced by TLR3 activation (Figure 7). Furthermore, ISG56 was upregulated in the synovial joint cells of patients with RA. These results indicate that ISG56 plays a role in RA pathogenesis. Thus, ISG56 may be a potential target for the development of novel RA therapies.

## Data Availability

The original contributions presented in the study are included in the article/supplementary material, further inquiries can be directed to the corresponding author.
